# Trans-cuff budesonide delivery and its effects on endotracheal intubation-induced subglottic injury

**DOI:** 10.3389/fped.2026.1886065

**Published:** 2026-09-29

**Authors:** Yi Zou, Qing Hu, Xiao Yan, Zhenli Lv, Jun Deng, Lili Zhong, Han Huang, Gaoyin Kong, Zaisheng Qin

**Affiliations:** 1 Department of Anesthesiology, Nanfang Hospital, Southern Medical University, Guangzhou, China; 2 Department of Anesthesiology, Hunan Provincial People's Hospital (The First Affiliated Hospital of Hunan Normal University), Changsha, China; 3 Department of Pathology, Hunan Provincial People's Hospital (The First Affiliated Hospital of Hunan Normal University), Changsha, China; 4 Department of Pharmacy, Hunan Provincial People's Hospital (The First Affiliated Hospital of Hunan Normal University), Changsha, China; 5 Hunan Provincial Key Laboratory of Pediatric Respirology, Pediatric Medical Center, Hunan Provincial People's Hospital (The First Affiliated Hospital of Hunan Normal University), Changsha, China

**Keywords:** budesonide, endotracheal intubation, intracuff, rabbits, subglottic

## Abstract

**Objectives:**

This study aimed to determine whether budesonide inhalation suspension (BIS) diffuses through polyvinyl chloride (PVC) endotracheal tube (ETT) cuffs and whether intracuff budesonide attenuates the acute subglottic injury during endotracheal intubation (ETI) in a rabbit model.

**Methods:**

*in vitro*, high-performance liquid chromatography was used to quantify trans-cuff diffusion of budesonide from 2.5-mm-ID PVC ETT cuffs. For the *in vivo* study, 32 New Zealand White rabbits were allocated to four groups (*n*=8): control (Con), air-inflated cuff (Air), normal saline-inflated cuff (NS), and BIS-inflated cuff (Bud). Intubated rabbits underwent 12 h of ETI at an intracuff pressure of 22 mmHg. Subglottic tissues were harvested immediately after extubation for histological and immunofluorescence analyses.

**Results:**

Budesonide diffusion across PVC ETT cuffs was temperature-dependent during the first 12 h and continued at 37°C for at least 14 days. *In vivo*, all ETI groups developed acute subglottic histological injury. Compared with the Air and NS groups, the Bud group had lower IL-1β, IL-6, and TNF-α fluorescence intensities (*P* < 0.05), whereas the measured histological outcomes and CD86/CD206 levels did not differ significantly among the ETI groups. No significant differences were observed between the Air and NS groups.

**Conclusions:**

Budesonide diffuses across PVC ETT cuffs, and intracuff BIS was associated with lower subglottic IL-1β, IL-6, and TNF-α fluorescence intensities during a 12-hour acute ETI model. The findings do not establish prevention of histological injury and require confirmation in longer-duration pharmacokinetic and outcome studies.

## Introduction

Endotracheal intubation (ETI) is widely utilized in clinical practice. The endotracheal tube (ETT) cuff, an essential component, is designed to prevent aspiration and air leakage. In pediatric populations, cuffed ETTs have gained preference over uncuffed counterparts due to the demonstrated advantages including reduced air leakage, lower reintubation rates, and decreased short-term complications (1–3). However, the unique laryngotracheal anatomy predisposes children to inadvertent positioning of the ETT cuff within the subglottic region (from the vocal cords to the cricoid cartilage) (4). The cricoid cartilage, being a non-expansile rigid ring, creates substantial mechanical stress when it is juxtaposed with an inflated cuff. This sustained pressure may induce mucosal compression, potentially progressing to ischemic necrosis. This pathophysiological cascade is recognized as a critical etiological factor in subglottic injury and subsequent development of subglottic stenosis (SGS) (5, 6).

SGS is a debilitating complication arising from fibroproliferative remodeling of the subglottic tissue and is strongly associated with prolonged ETI (6). Central to its pathogenesis is an excessive and dysregulated inflammatory response triggered by mechanical trauma, which drives pathological collagen deposition and scar formation (7). Furthermore, given the high recurrence propensity of SGS, its clinical management remains considerably challenging (8), making prevention of SGS formation critically important. Pre-emptive anti-inflammatory interventions have demonstrated efficacy in attenuating fibrotic cascades, suggesting that early modulation of inflammatory pathways may mitigate scar-related sequelae (9). Therefore, initiating anti-inflammatory therapy concurrently with prolonged ETI may provide clinical benefits to patients.

Corticosteroids represent the most widely utilized anti-inflammatory medications for respiratory diseases, with multiple administration routes, including oral ingestion, intravenous injection, inhalation, topical application, and intralesional injection (10, 11). During ETI, corticosteroids are commonly administered intravenously or via inhalation. However, intravenous administration carries systemic side effects (10), while inhaled delivery primarily targets the bronchi and lungs which fails to provide adequate anti-inflammatory coverage at the subglottic contact points between the ETT cuff and tracheal mucosa. Although clinical evidence demonstrates that topical corticosteroid coating on ETTs can prevent postoperative sore throat (12), this effect is transient owing to rapid drug absorption. While drug-eluting ETTs represent a promising alternative (13), their high cost limits widespread clinical application. Therefore, more appropriate approaches need to be explored.

Accumulating clinical evidence demonstrates that intracuff lidocaine administration effectively reduces sore throat in patients undergoing ETI under general anesthesia (14). This therapeutic effect is mechanistically supported by *in vitro* studies confirming lidocaine diffusion through polyvinyl chloride (PVC) cuff membranes, enabling direct pharmacologic action on the tracheal mucosa (15). Therefore, if corticosteroids could diffuse through the cuff, they would provide sustained anti-inflammatory effects at the tracheal-cuff interface. While previous clinical trials reported that intracuff dexamethasone alleviated post-extubation symptoms (sore throat, hoarseness, and cough) in patients undergoing general anesthesia with ETI, these studies provided no *in vitro* evidence of dexamethasone diffusion through ETT cuff material (16, 17). Furthermore, intralesional triamcinolone acetonide injection has demonstrated clinical efficacy in SGS management (18), suggesting its potential as an alternative. However, our preliminary experiments did not detect measurable diffusion of either dexamethasone or triamcinolone acetonide across the ETT cuff membrane.

Budesonide inhalation suspension (BIS) is a widely used inhaled corticosteroid. Clinical investigations have established budesonide's therapeutic benefits across multiple airway pathologies, including sore throat (19), acute infectious laryngitis (20), post-intubation laryngeal granuloma (21), and postoperative tracheal tissue granulation (22). Interestingly, our preliminary observations indicated detectable diffusion of budesonide across the ETT cuff.

Based on these observations and evidence, we hypothesized that intracuff budesonide would permit sustained trans-cuff delivery and attenuate the acute subglottic injury induced by the ETT cuff. To our knowledge, relevant evidence remains limited. We therefore quantified budesonide diffusion kinetics *in vitro* and tested whether intracuff budesonide altered acute subglottic histological injury and inflammatory markers during 12 h of ETI in rabbits.

## Materials and methods

### *In vitro* drug diffusion study

Six cuffed PVC ETTs with an internal diameter (ID) of 2.5 mm (Henan Tuoren Kingtaek Medical Device Co., Ltd., China) were prepared. Each cuff was inflated with 500 μg/mL BIS (Pulmicort Respules®, AstraZeneca Pty Ltd., North Ryde, Australia) and adjusted to an intracuff pressure (ICP) of 22 mmHg (30 cmH_2_O) using an invasive pressure monitoring system connected with a patient monitor (GE Dash 4000, General Electric Co., USA) (23). Each ETT was immersed individually in 30 mL of phosphate-buffered saline (PBS) in glass bottles (15). Three of the bottles were incubated in a 37  °C water bath for 12 h (Group HT, representing human body temperature), while the other three were incubated in a 38.5  °C water bath for 12 h (Group RT, representing rabbit body temperature).

During the initial 12-hour water bath incubation, 500 μL aliquots were collected hourly from both the HT and RT groups. After completion of the initial 12-hour incubation, the three bottles in Group HT were transferred to a 37 °C incubator and continuously observed until day 14. Additional samples were collected once daily from day 1 to day 14 in Group HT. Immediately after each daily sample collection, the PBS in each glass bottle was completely replaced with 30 mL of fresh 4 ℃ PBS. The ICP was measured concurrently and restored to 22 mmHg by adding BIS. For the first 24 h, cumulative budesonide diffusion was calculated based on the budesonide concentration in the PBS, with correction for the amount removed during each 500 μL hourly sampling. Thereafter, the amount of budesonide recovered during each daily interval was added to the cumulative amount from the preceding intervals to obtain the cumulative amount of diffused budesonide. All samples were stored immediately at 4 °C until analysis. Budesonide concentrations in all collected samples were quantified by high-performance liquid chromatography (HPLC) using the method described below. Detailed schematics of the ICP measurement system and ETT immersion methodology are provided in Figure 1.

**Figure 1:**
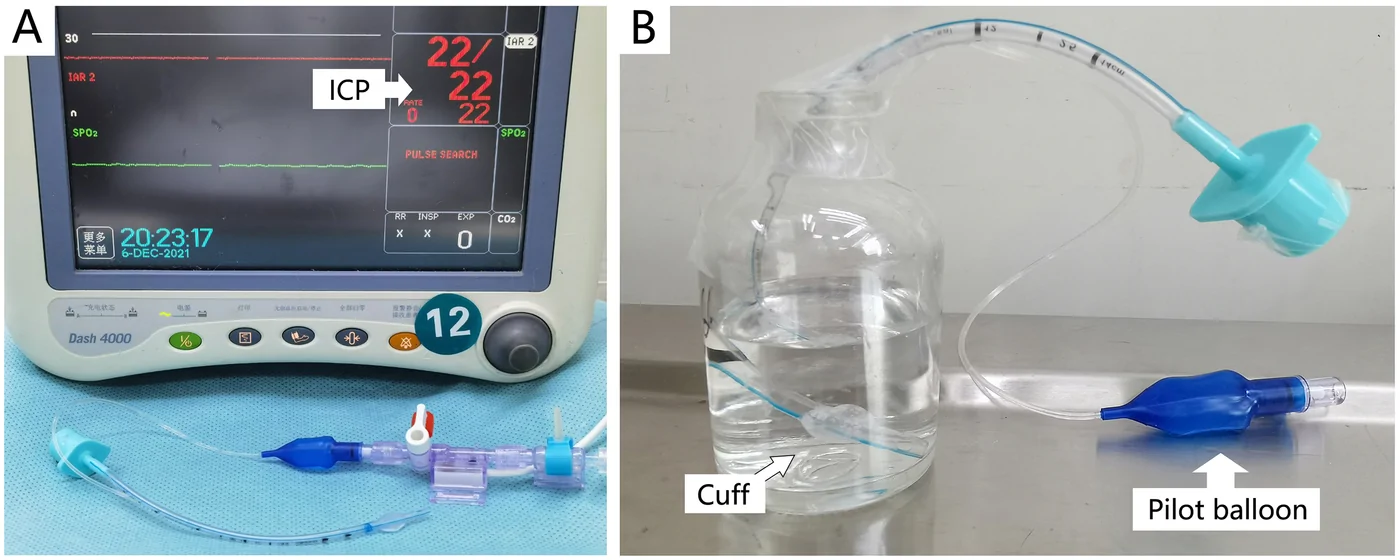
The ICP was measured using an invasive pressure monitoring system **(A)**; the ETT cuff was immersed in PBS **(B)**. ICP, intracuff pressure; PBS, phosphate-buffered saline.

### HPLC for budesonide concentration test

HPLC was performed using a reversed-phase C18 column (Xtimate C18, 4.6 × 250 mm, 5 μm, Agilent 1260 Infinity HPLC, Agilent Technologies, Inc, USA). The mobile phase consisted of methanol and H_2_O (72:28, v/v). The flow rate was 1.0 mL/min at 35 °C. Ultraviolet detection was performed at 240 nm. The budesonide standard (National Institutes for Food and Drug Control, China) was diluted to 5, 1, 0.5, 0.1, 0.05, 0.01, and 0.005 μg/mL. These solutions were analyzed to establish the relationship between budesonide concentrations and peak areas.

### *In vivo* animal experiment

#### Experimental animals

This study was approved by the Animal Ethics Committee of Hunan Provincial People's Hospital (No. 2021–07). Thirty-two male New Zealand White rabbits (2.2–2.9 kg; Hunan Peace Biotechnology Co., China) were acclimatized for 3 days under 12/12-hour light/dark cycles with free access to water and standard chow prior to experimental procedures.

#### Anesthesia and ETI protocol

Rabbits were pretreated intramuscularly with midazolam 0.5 mg/kg (Jiangsu Nhwa Pharmaceutical Co., Ltd., China) and sufentanil 0.5 μg/kg (Yichang Humanwell Pharmaceutical Co., Ltd., China) administered into the lumbar epaxial muscles. A 24-gauge indwelling catheter was aseptically placed in the left marginal ear vein for sequential anesthetic induction using sufentanil 0.1–0.2 μg and propofol 2–4 mg (Jiangsu Yingkang Biopharmaceutical Co., Ltd., China). Animals were positioned in supine recumbency with neck extension on a heating pad (Beijing Zhongke Huida Technology Co., Ltd. China). Under video-otoscope (24) (Teacher Yang Bo E-Commerce Co., Ltd., China), the glottis was visualized, and an ID 2.5 mm cuffed ETT was advanced into the trachea under direct visualization (Figure 2A).

**Figure 2:**
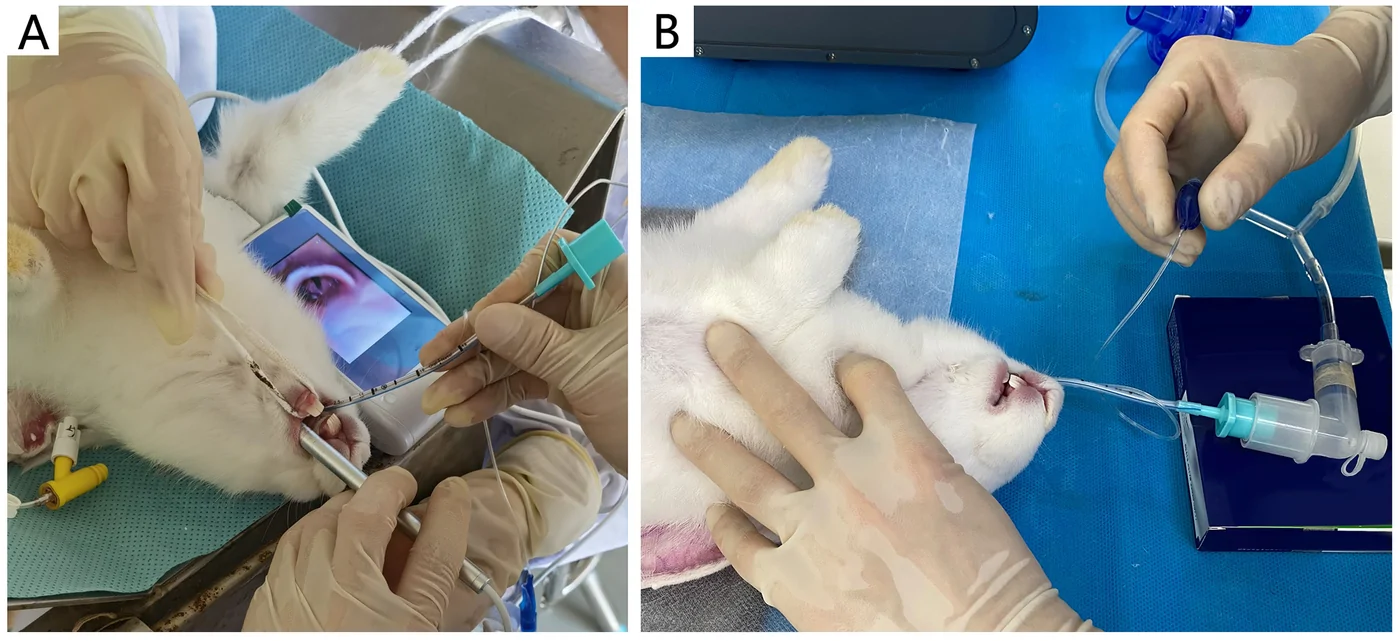
Intubation using a video-otoscope **(A)** cuff position confirmation **(B).**

Rabbits were randomly allocated into four experimental groups (*n* = 8 per group):

Group Con: Rabbits underwent glottic exposure without ETI, returned to housing for 12 h prior to euthanasia.
Group Air: ETT cuff inflated with air to ICP 22 mmHg.
Group NS: ETT cuff inflated with normal saline (NS) to ICP 22 mmHg.
Group Bud: ETT cuff inflated with BIS to ICP 22 mmHg.

In the ETI groups, following intravenous administration of cisatracurium 0.2 mg/kg (Jiangsu Hengrui Pharmaceuticals Co., Ltd., China), animals were maintained in the supine position and mechanically ventilated (HX-101E ventilator, Taimeng Software Co., China) for 12 h. Mechanical ventilation was adjusted to a tidal volume of 10–16 mL/kg, an inspiratory-to-expiratory ratio of 2:3, and a respiratory rate of 40–60 breaths/min. Anesthesia was maintained with midazolam at 0.4 mg/kg/h, sufentanil at 1.2 μg/kg/h, and cisatracurium at 0.4 mg/kg/h. The anesthetic agents were diluted in compound sodium chloride injection (Hunan Jinjian Pharmaceutical Co., Ltd., China) and administered by continuous intravenous infusion, with the total infusion rate adjusted between 8 and 20 mL/h (25–27). The ETT cuff was positioned within the subglottic region, confirmed via palpation of the cricothyroid membrane and pilot balloon compression (Figure 2B). ICP, pulse rate (PR), oxygen saturation (SpO₂), end-tidal carbon dioxide (P_ET_CO₂), mean arterial pressure (MAP), and rectal temperature (T) were monitored hourly following ETI. When the measured ICP deviated from the target value of 22 mmHg, the cuff pressure was adjusted by inflation or deflation using either air, NS, or BIS.

The duration of ETI was set at 12 h based on preliminary diffusion experiments and previous reports. Preliminary testing demonstrated detectable budesonide outside the cuff after approximately 3–4 h, while previous clinical data suggested that intranasal budesonide could exert pharmacological effects within approximately 7 h (28). Therefore, a 12-hour ETI period was selected to allow sufficient trans-cuff budesonide exposure and to establish a reproducible acute subglottic injury model.

At the end of the 12-hour experimental period, while the rabbits remained under general anesthesia, euthanasia was performed by intravenous administration of sodium pentobarbital (100 mg/kg) through the marginal auricular vein. Death was confirmed by the absence of heartbeat, spontaneous respiration, corneal reflex, and response to noxious stimulation. After confirmation of death, the laryngotracheal complex was surgically exposed, the cuff was deflated, and the ETT was removed. The larynx and five contiguous subglottic tracheal cartilage rings were entirely resected and fixed in 4% formalin. After fixation, specimens were transversely sectioned at the level of the cricoid cartilage to an appropriate thickness and embedded in paraffin.

### Histological analysis

The tissue slices (4*μ*m) were stained with hematoxylin and eosin (H&E) for histopathological evaluation. The images of H&E stained sections were captured after scanning. First, the proportional extent of mucosal injury in the cricoid cartilage (defined as the ratio of damaged mucosal circumference to total mucosal circumference) was compared. Second, the thickness of the lamina propria embedded with cartilage was measured in four randomly selected 100× fields per section. This thickness was defined as the distance from the cartilage surface to the most superficial layer of the lamina propria. Measurements were obtained using ImageJ software (National Institutes of Health, Bethesda, MD, USA), and the mean value of the four fields was calculated and used for each section in statistical analyses. Third, inflammatory status was evaluated in the sections through three distinct scoring dimensions: degree of inflammation (0-none, 1-mild-scattered cells in lamina propria, 2-moderate, 3-severe-fills most the area affected), inflammatory involvement (0-none, 1-up to 1/3 of section, 2–1/3–2/3 of section, 3–2/3 to entire section), and mucosal ulceration (0-none, 1-focal mild ulcer, 2-multiple small ulcers or a single, moderately sized ulcer, 3-large areas of ulceration in section) (29). Neutrophil infiltration was quantified by manual counting of neutrophils in four randomly selected 400 × fields per tissue section (30). The average count from these four fields was calculated and used as the representative neutrophil count for each section in subsequent statistical analysis.

### Immunofluorescence staining and semi-quantitative analysis

Tyramide signal amplification (TSA)-based immunofluorescence staining was performed on paraffin-embedded subglottic tissue sections (4 μm). After dewaxing, rehydration, and heat-induced antigen retrieval in EDTA buffer (pH 9.0), sections were treated with 3% H₂O₂ to block endogenous peroxidase activity.

For detection of inflammatory cytokines, sections were incubated overnight at 4 °C with mouse anti-IL-1β (66737–1-Ig, Proteintech Group, Inc., Wuhan, China), mouse anti-IL-6 (66146–1-Ig, Proteintech Group, Inc., Wuhan, China), or mouse anti-TNF-α (60291–1-Ig, Proteintech Group, Inc., Wuhan, China). After incubation with HRP-conjugated anti-mouse secondary antibody, TSA-570 fluorophore was applied for signal amplification. Nuclei were counterstained with DAPI.

For double immunofluorescence staining of CD86 and CD206, sections were first incubated overnight at 4 °C with mouse anti-CD86 antibody (68674–2-Ig, Proteintech Group, Inc., Wuhan, China), followed by HRP-conjugated anti-mouse secondary antibody and TSA-570 amplification. After heat-mediated antibody stripping, sections were incubated with mouse anti-CD206 antibody (60143–1-Ig, Proteintech Group, Inc., Wuhan, China), followed by HRP-conjugated anti-mouse secondary antibody and TSA-520 amplification. Nuclei were counterstained with DAPI.

Negative controls were performed by omitting primary antibodies. Fluorescence images were acquired using a confocal laser scanning microscope at 400× magnification. For each section, four randomly selected fields were analyzed. The mean fluorescence intensity (MFI) of each target protein was quantified using ImageJ software (National Institutes of Health, Bethesda, MD, USA). The MFI value of each sample was calculated as the average value of four fields, yielding eight independent values per group (*n* = 8). Relative fluorescence intensity was calculated by dividing the MFI of each sample by the mean MFI of the Con group. These relative fluorescence intensity values were used for statistical analysis.

All histological and immunofluorescence analyses were performed by investigators blinded to the experimental group allocation.

### Statistical analysis

Statistical analyses were performed using GraphPad Prism 10 (GraphPad Software, San Diego, CA, USA). Data are presented as mean ± standard deviation (SD) or median (interquartile range, IQR). Cumulative budesonide diffusion within 12 h was analyzed using two-way repeated-measures ANOVA followed by Sidak's multiple-comparisons test. The 14-day cumulative diffusion data were fitted using a one-phase exponential association model. Vital signs and ICP were compared using two-way repeated-measures ANOVA with Sidak's *post-hoc* test. Histological and immunofluorescence data were analyzed using one-way ANOVA with Tukey's *post-hoc* test or Kruskal–Wallis test with Dunn's *post-hoc* test as appropriate. A *P* value < 0.05 was considered statistically significant.

## Results

### *In vitro* drug diffusion study

As shown in Figure 3A, the budesonide standard exhibited a peak retention time of 9.979 min (arrow).

**Figure 3:**
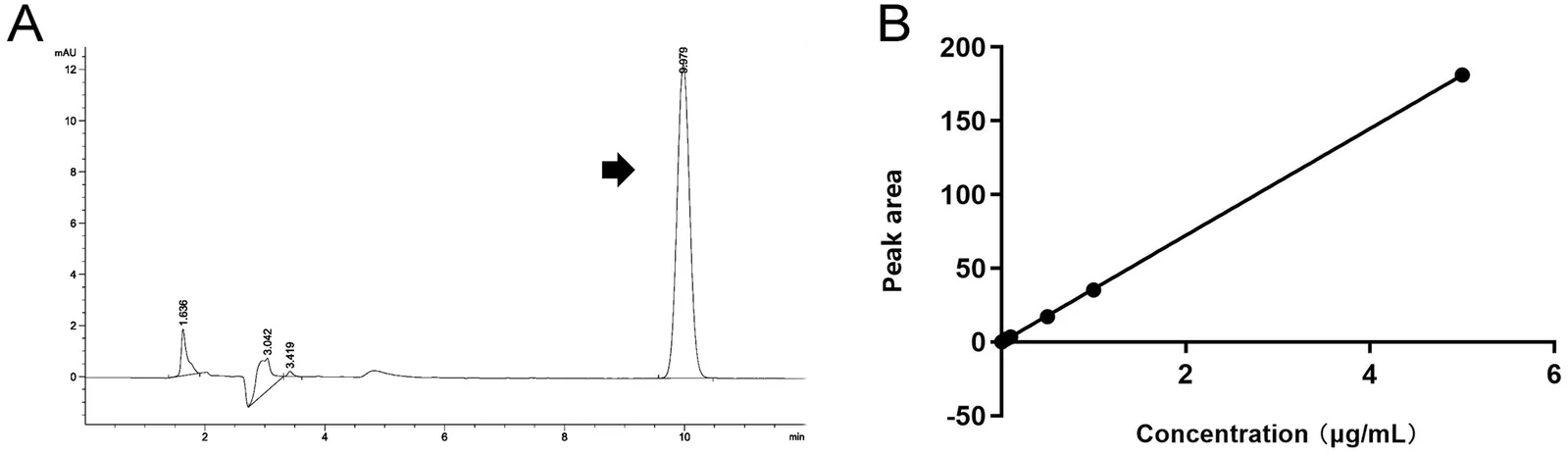
The retention peak of the budesonide standard (arrow) **(A)** and the standard curve of budesonide in the concentration range of 0.005–5.0 μg/mL **(B).**

As shown in Figure 3B, the peak area of budesonide exhibited a good linear relationship with its concentration over the range of 0.005–5.0 μg/mL. Fitted by simple linear regression, the equation of the standard curve was Y = 36.26X - 0.2758. This linear relationship was highly significant (*F* = 95,202, *P* < 0.001), with a coefficient of determination (*R*^2^) of 0.9999, indicating that 99.99% of the variation in peak area could be explained by the concentration. The 95% confidence intervals for the slope and intercept were 35.96 to 36.56 and −0.8609 to 0.3093, respectively. This standard curve met the requirements for quantitative analysis and could be used for the calculation of budesonide concentration in subsequent samples.

As shown in Figure 4A, the budesonide diffusion amount increased progressively with duration of cuff inflation with BIS (*F* = 2,238, *P* < 0.001). Temperature exerted a significant effect on cumulative budesonide diffusion, with the cumulative amount of budesonide diffused from the ETT cuff in the RT group (maintained at 38.5℃) being significantly greater than that in the HT group (maintained at 37℃) (*F* = 41.5, *P* = 0.023). Budesonide diffusion was detected in all ETT cuffs of both groups at 3 h after intracuff budesonide injection. *Post-hoc* analysis revealed that from 4 to 12 h, the total diffusion amount in the RT group remained consistently higher than that in the HT group (*P* < 0.05).

**Figure 4:**
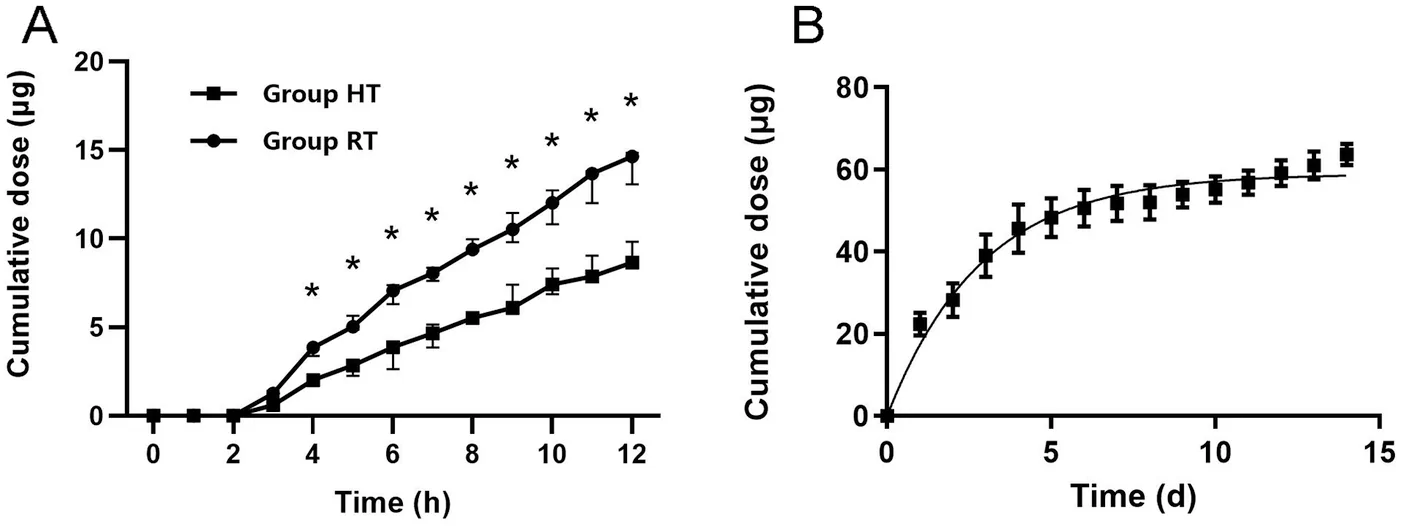
The cumulative amounts of budesonide diffused over 12 h **(A)** and 14 days **(B)**. Data are presented as mean ± SD. *Compared with group HT at the same time point, *P* < 0.05.

As shown in Figure 4B, the cumulative amount of budesonide diffused across the ETT cuff increased progressively throughout the 14-day observation period, demonstrating sustained trans-cuff diffusion. The cumulative diffusion data were fitted using a one-phase exponential association model (R^2^ = 0.9438), yielding an estimated plateau of 58.99 μg (95% CI: 56.73–61.51) and a rate constant K of 0.3475 (95% CI: 0.2972–0.4069). The model-estimated time to reach 50% of the plateau was 2 days. Notably, cumulative budesonide diffusion continued to increase through day 14. During the 14-day period, no turbidity changes were observed in the PBS in any of the three glass bottles, and no cuff rupture occurred.

### *In vivo* animal experiment

Body weight was comparable among the four groups, whereas the number of ETI attempts and ETI depth were comparable among the three ETI groups (*P* > 0.05, Figure 5). Figure 6 presents the comparative physiological profiles, including vital signs and ICP, across ETI groups during anesthetic maintenance. No statistically significant intergroup variations were detected in PR, SpO₂, MAP, P_ET_CO₂, T or ICP (*P* > 0.05).

**Figure 5:**
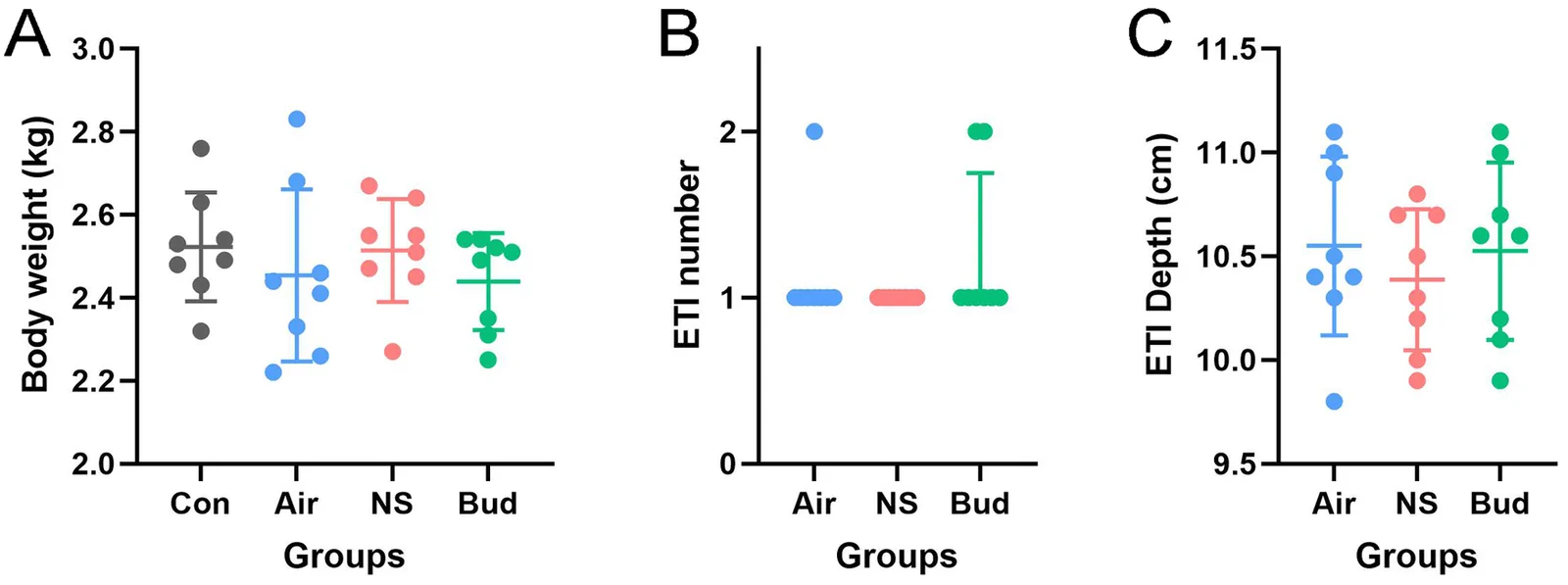
Characteristics of the rabbits. ETI, endotracheal intubation. Data are median with IQR **(A,B)** or mean with SD **(C).**

**Figure 6:**
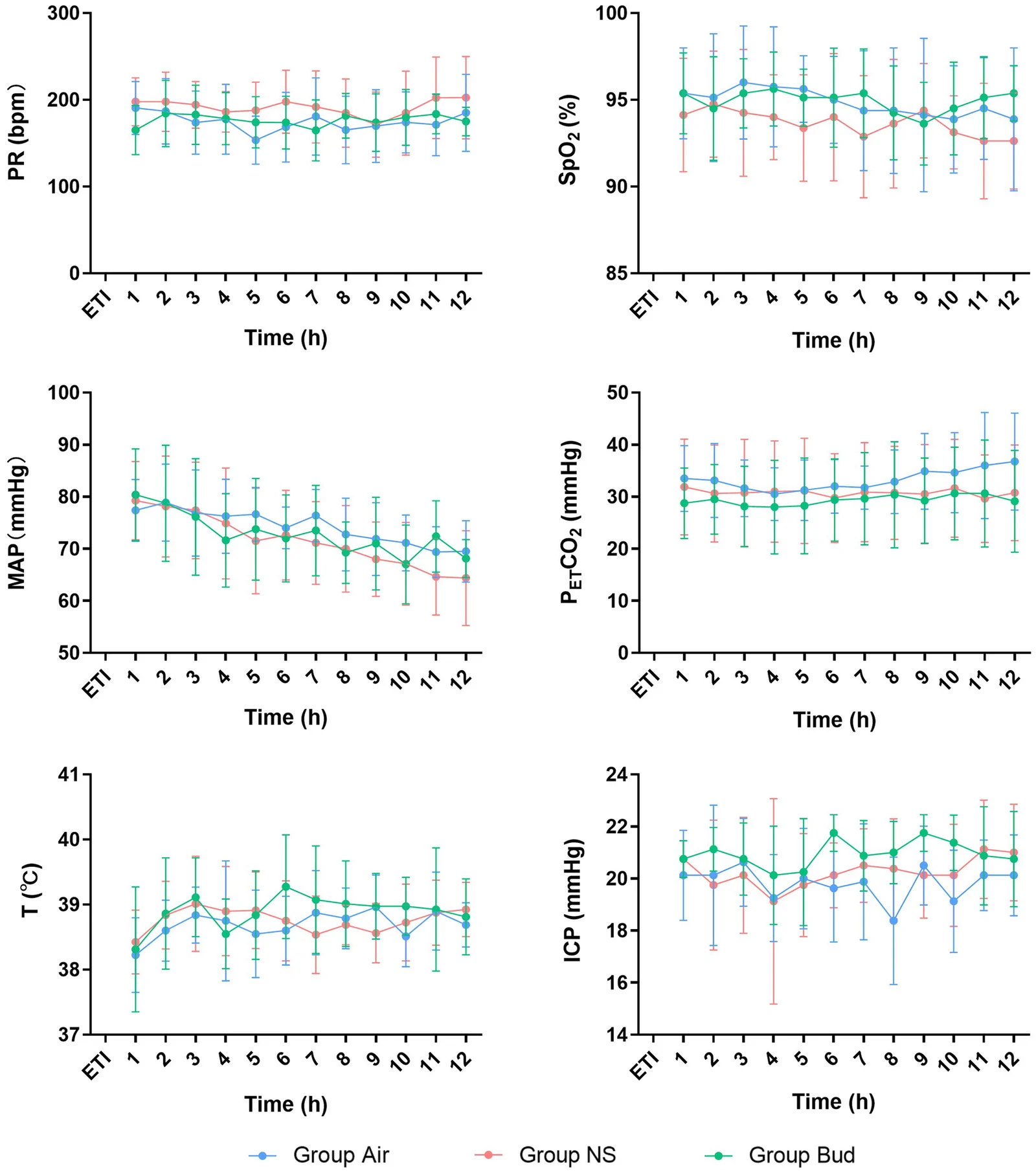
The profiles of vital signs and ICP of the ETI rabbits during anesthesia. Data are presented as mean ± SD. ICP, intracuff pressure; ETI, endotracheal intubation.

### Histological findings

Histological examination of the subglottic tissue in Group Con revealed intact architecture with clearly identifiable mucosa, lamina propria, and cartilage. Scattered lymphocytes and only occasional neutrophils were observed in the lamina propria (Figure 7A). In contrast, ETI rabbits showed significant subglottic injury characterized by: (1) mucosal epithelial damage with ciliary loss and disruption of columnar cells and basement membrane; (2) acute inflammatory changes in both mucosa and lamina propria, evidenced by neutrophil infiltration (Figure 7B, C, D); and (3) prominent ulceration and hemorrhage.

**Figure 7:**
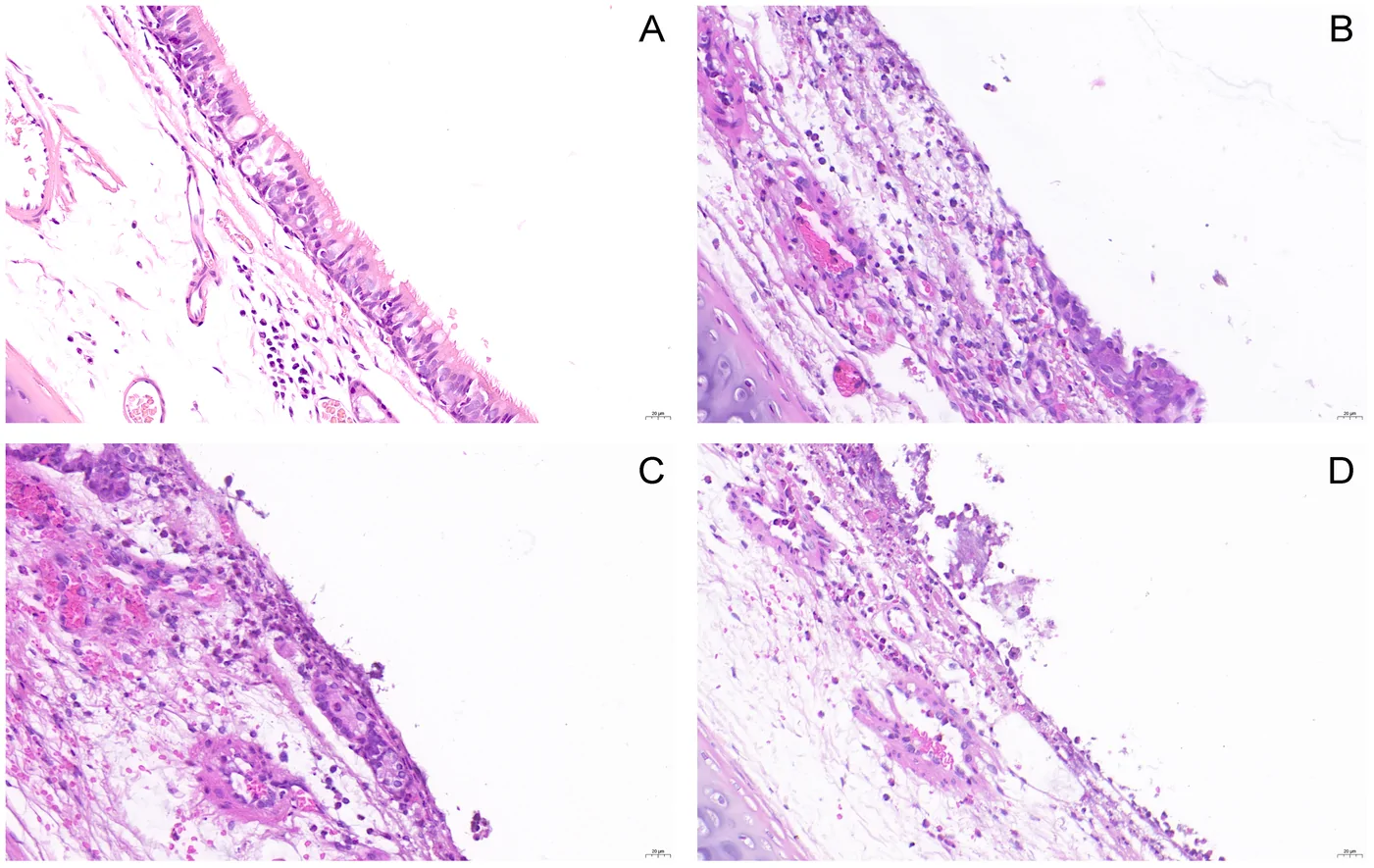
Representative histological views of the subglottic mucosa and lamina propria. **A**: group Con; **B**: group Air; **C**: group NS; **D**: group Bud. Magnification×400.

As shown in Figure 8, compared with the Con group, all ETI groups showed significant subglottic mucosal damage (Figure 8A, *P* < 0.001), reduced lamina propria thickness (Figure 8B, *P* < 0.01), and higher inflammation scores (Figure 8C, *P* < 0.05). The Air and NS groups also had higher mucosal ulceration scores (Figure 8D, *P* < 0.01), inflammatory involvement scores (Figure 8E, *P* < 0.01), and neutrophil counts (Figure 8F, *P* < 0.01) than the Con group. None of the measured histological outcomes differed significantly between the Bud group and the Air or NS group.

**Figure 8:**
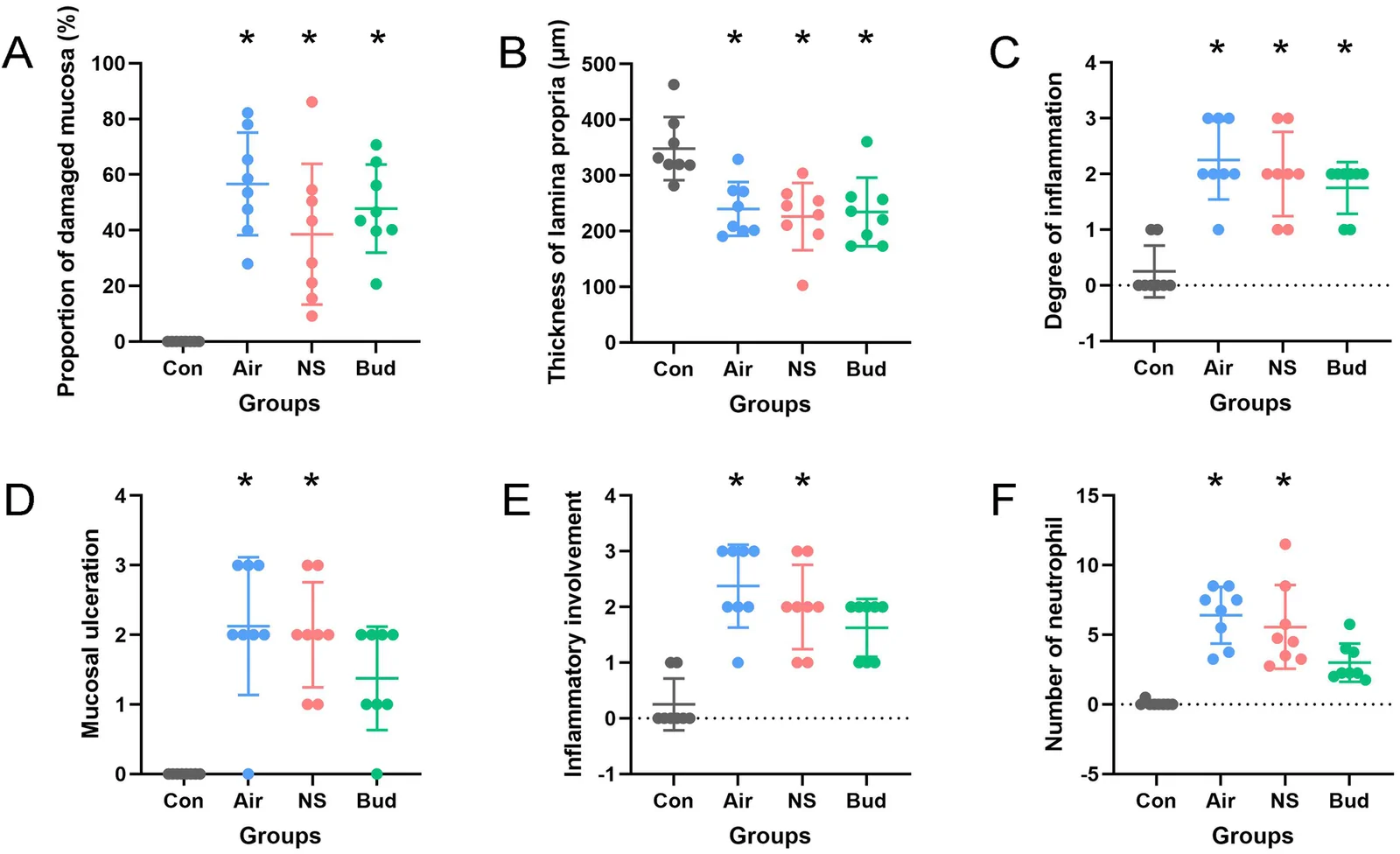
**A**: mucosal damage ratio (%); **B**: lamina propria thickness (μm); **C**: degree of inflammation score; **D**: mucosal ulceration score; **E**: inflammatory involvement score; **F**: neutrophil count per high-power field. Data are presented as mean ± SD **(A,B)** or median with IQR **(C–F)**. *Compared with group Con, *P* < 0.05.

### Immunofluorescence staining

As shown in Figure 9, immunofluorescence-positive signals for IL-1β, IL-6, and TNF-α were predominantly localized in the mucosal and submucosal layers of the subglottic tissue in all experimental groups. Semi-quantitative analysis revealed that the relative fluorescence intensities of IL-1β, IL-6, and TNF-α were significantly increased in both the Air and NS groups compared with the Con group (*P* < 0.001). In contrast, the Bud group showed significantly reduced relative fluorescence intensities of IL-1β, IL-6, and TNF-α compared with the Air and NS groups (*P* < 0.05). Although IL-1β and IL-6 levels in the Bud group remained higher than those in the Con group (*P* < 0.01), no significant difference in TNF-α fluorescence intensity was observed between the Bud and Con groups (*P* = 0.242).

**Figure 9:**
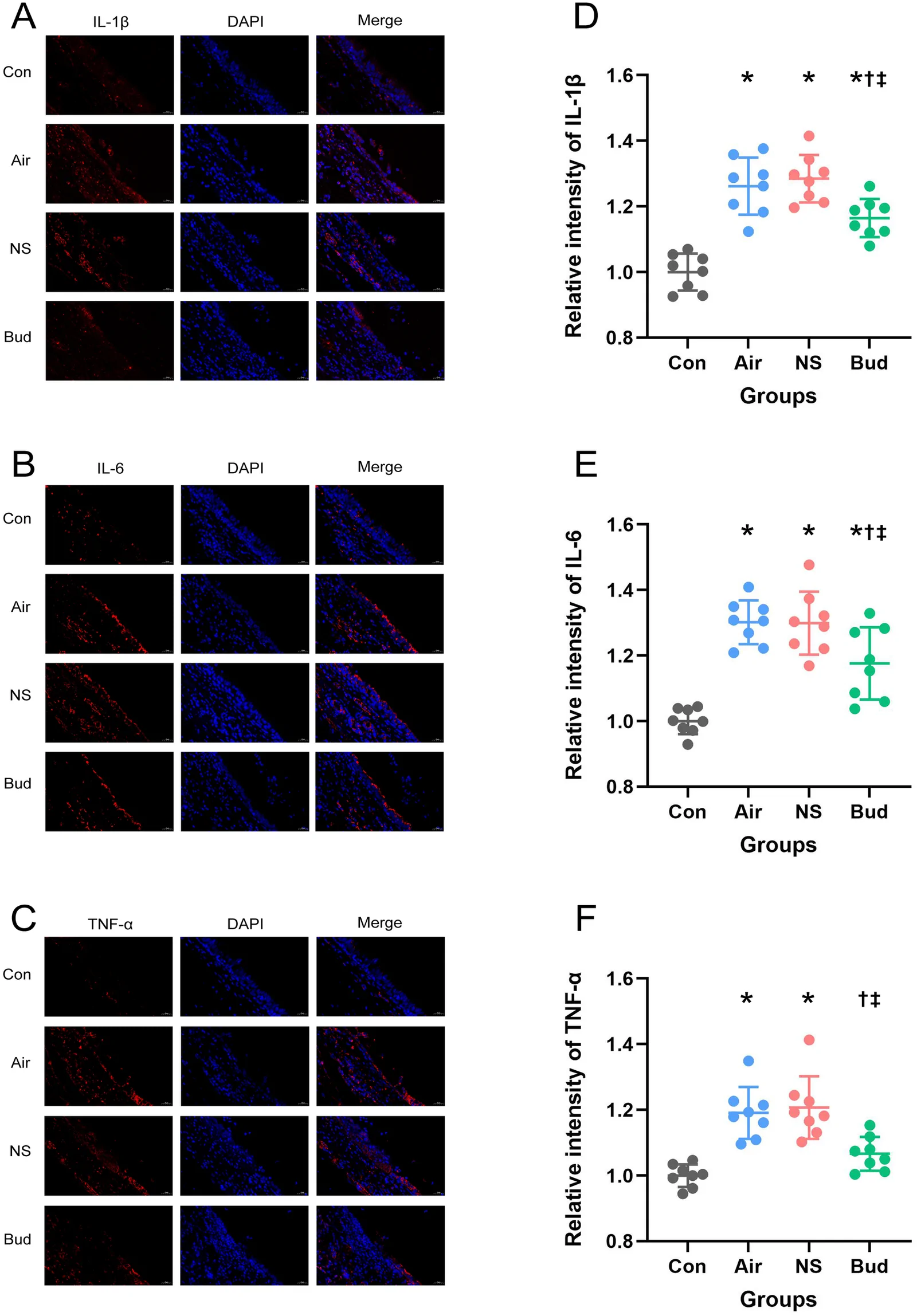
Representative immunofluorescence images of IL-1β **(A)**, IL-6 **(B)**, and TNF-α **(C)** magnification: ×400. Quantification of relative fluorescence intensity of IL-1β **(D)**, IL-6 **(E)**, and TNF-α **(F)** Relative fluorescence intensity was calculated as the ratio of the MFI of each sample to the mean MFI of the Con group. MFI: mean fluorescence intensity. *P < 0.05 compared with the Con group; †*P* < 0.05 compared with the Air group; ‡*P* < 0.05 compared with the NS group.

As shown in Figure 10, CD86 and CD206 positive signals were predominantly localized in the mucosal and submucosal layers of the subglottic tissue in all experimental groups. Semi-quantitative analysis based on relative fluorescence intensity demonstrated that the Air, NS, and Bud groups exhibited significantly increased CD86 and CD206 fluorescence intensities compared with the Con group (*P* < 0.05). No significant differences were observed among the Air, NS, and Bud groups (*P* > 0.05).

**Figure 10:**
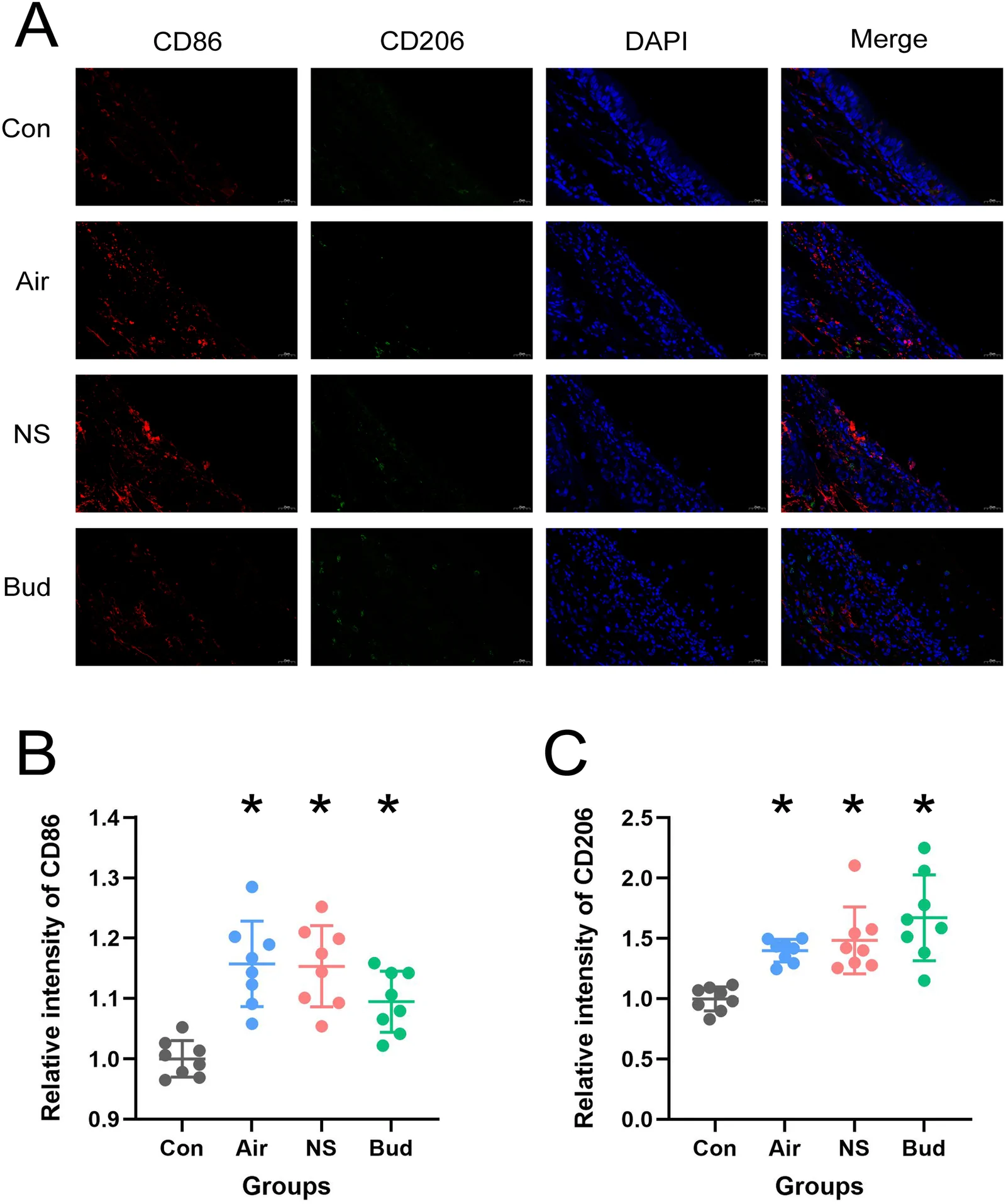
Representative double immunofluorescence images of CD86 (red) and CD206 (green) **(A)** magnification: ×400. Quantification of relative fluorescence intensity of CD86 **(B)** and CD206 **(C)** Relative fluorescence intensity was calculated as the ratio of the MFI of each sample to the mean MFI of the Con group. MFI: mean fluorescence intensity. *P < 0.05 compared with the Con group.

No significant differences were detected between the Air and NS groups in any histological or immunofluorescence parameters (*P* > 0.05).

## Discussion

The main findings of this study were that budesonide could diffuse across PVC ETT cuffs and that intracuff budesonide reduced the expression of IL-1β, IL-6, and TNF-α after 12 h of ETI. However, no significant improvement was observed in the histological injury parameters, and CD86 and CD206 expression was unchanged among the intubated groups. Therefore, the present findings indicate an early local anti-inflammatory effect rather than prevention of subglottic injury.

Budesonide became detectable outside the cuff after 3–4 h and continued to diffuse for at least 14 days *in vitro*. The greater cumulative budesonide diffusion observed at 38.5 °C than at 37 °C is consistent with the temperature-dependent diffusion previously described for intracuff lidocaine (15). The ID 2.5 mm ETT inflated with BIS demonstrated sustained trans-cuff budesonide diffusion, with a cumulative amount approaching 60 μg over 14 days, supporting the feasibility of this approach for prolonged local drug delivery. The slower increase in cumulative diffusion after 24 h may be related to changes in experimental conditions, including the transition from a water bath to an incubator, daily replacement of PBS, and possible adsorption of BIS particles onto the inner cuff surface, all of which may reduce the apparent diffusion rate.

Although the amount of budesonide diffused was relatively low (approximately 0.7 μg/h during the first 12 h), only approximately 14%–16% of the nominal dose of nebulized budesonide has been shown to deposit in the lungs in healthy adults (31). Together with the reported onset of action of intranasal budesonide within approximately 7 h (28), these findings support the possibility that relatively limited local exposure may modulate early inflammatory responses.

In the *in vivo* study, rabbits were selected as the animal model because of their comparable laryngotracheal anatomy to that of young children and their established use in laryngotracheal injury models (5, 29, 32). This model was considered appropriate for investigating cuff-induced subglottic injury following ETI in young children. Our study demonstrated that 12-hour ETI in rabbits induced significant subglottic injury, while no significant difference in H&E-based inflammation scores was observed between the Air and NS groups. Although intracuff budesonide did not significantly reduce the histological inflammation scores, significantly lower IL-1β, IL-6, and TNF-α fluorescence intensities were observed in the Bud group. These findings suggest that the anti-inflammatory effect of intracuff budesonide may emerge at the molecular level before detectable histological improvement occurs.

Previous studies have demonstrated that locally delivered budesonide can exert anti-inflammatory effects in different inflammatory models. In a rat model of inflammatory bowel disease, locally administered budesonide-loaded nanoparticles markedly suppressed colonic TNF-α expression, reduced inflammatory cell infiltration, and promoted mucosal repair and ulcer healing (33). Notably, the cumulative budesonide amount released over 48 h in that study was less than 10 μg, suggesting that localized delivery may achieve biological effects with limited drug exposure. Similarly, in a rabbit model of endotoxin-induced acute respiratory distress syndrome, intratracheal administration of budesonide (0.5 mg/kg) rapidly reduced inflammatory cytokines, including TNF-α, IL-1β, and IL-8, within 1 h after administration (34). These studies suggest that local budesonide exposure can produce measurable anti-inflammatory effects within a relatively short period.

The reduction in subglottic IL-1β, IL-6, and TNF-α fluorescence observed in the present study is consistent with the established anti-inflammatory actions of inhaled corticosteroids. Budesonide binds to intracellular glucocorticoid receptors (GRs), which translocate into the nucleus and regulate inflammatory gene expression through mechanisms including transrepression of pro-inflammatory transcription factors, thereby reducing the production of cytokines such as TNF-α (35). However, tissue budesonide concentrations were not measured in the present study; therefore, a direct concentration–response relationship between trans-cuff budesonide diffusion and the observed tissue inflammatory changes cannot be established.

CD86 and CD206 expression increased after ETI but did not differ among the three intubated groups. Because CD86 and CD206 expression alone cannot comprehensively characterize macrophage polarization, the present findings should be interpreted primarily as changes in these two inflammatory markers rather than as evidence of a shift in macrophage phenotype. Previous studies have reported modulation of macrophage polarization by carrier-based budesonide delivery in other inflammatory models (36). However, such effects may depend on the formulation, dose, tissue, and timing.

Intracuff administration could, in principle, concentrate drug delivery at the cuff-mucosa interface without requiring a modified ETT. No cuff rupture was observed in this series. Nevertheless, before clinical application, several issues require further evaluation, particularly cuff integrity, formulation compatibility, and the risk of leakage or aspiration. Pharmacokinetic studies are also needed to determine local tissue exposure and systemic absorption.

This study has several limitations. First, budesonide concentrations were not measured in subglottic tissue, cuff-adjacent fluid, or plasma, and the *in vitro* system cannot reproduce the biological barriers present *in vivo*. Second, only one 12-hour ETI duration and one immediate post-extubation time point were studied; different ETI durations and post-extubation intervals may yield different findings. Third, no significant improvement was detected in the measured histological injury outcomes, and fibrosis, stenosis, and long-term functional outcomes were not assessed. Finally, the modest sample size, use of only male rabbits, and species-specific airway anatomy should also be considered when interpreting the results.

## Conclusions

Intracuff budesonide allowed sustained trans-cuff diffusion of budesonide *in vitro* and reduced subglottic IL-1β, IL-6, and TNF-α expression after 12 h of ETI in rabbits. However, it did not significantly improve histological injury or alter CD86 or CD206 expression. These findings suggest that intracuff budesonide may attenuate the early local inflammatory response to ETI, although its effects on subsequent fibrosis or subglottic stenosis remain unknown. Longer-duration pharmacokinetic and outcome studies are required before clinical translation.

## Data Availability

The original contributions presented in the study are included in the article/Supplementary Material, further inquiries can be directed to the corresponding authors.
